# Functional assessment: Free thin anterolateral thigh flap versus free radial forearm reconstruction for hemiglossectomy defects

**DOI:** 10.4317/medoral.20727

**Published:** 2015-10-09

**Authors:** Mingxing Lu, Guowen Sun, Qingang Hu, Enyi Tang, Yujia Wang

**Affiliations:** 1 BDS. Attending Surgeon, Department of Oral and Maxillofacial Surgery, Nangjing Stomatological Hospital, Medical School of Nanjing University; 2 DDS, MD, PhD. Attending Surgeon, Department of Oral and Maxillofacial Surgery, Nangjing Stomatological Hospital, Medical School of Nanjing University; 3 DDS, MD, PhD. Professor, Department of Oral and Maxillofacial Surgery, Nangjing Stomatological Hospital, Medical School of Nanjing University; 4 MDS. Professor, Director, Department of Oral and Maxillofacial Surgery, Nangjing Stomatological Hospital, Medical School of Nanjing University; 5 MDS. Resident, Department of Oral and Maxillofacial Surgery, Nangjing Stomatological Hospital, Medical School of Nanjing University

## Abstract

**Background:**

To compare free thin anterolateral thigh (ALT) flap with free radial forearm (FRF) flap in the reconstruction of hemiglossectomy defects, and to introduce our methods and experience in the tongue reconstruction with free thin ALT flap.

**Material and Methods:**

The clinicopathologic data of 46 tongue carcinoma cases hospitalized from December 2009 to April 2014 were obtained from Nangjing Stomatological Hospital, Medical School of Nanjing University. All the subjects were evaluated for the articulation and the swallowing function 3 months after the surgery.

**Results:**

Among these 46 patients, 12 patients underwent tongue reconstruction after hemiglossectomy with ALT flap; 34 patients underwent tongue reconstruction with FRF flap. The differences in the incidence of vascular crisis, the speech and the swallowing function between two groups were not significant (*P*>0.05).

**Conclusions:**

Thin ALT flap could be one of the ideal flaps for hemiglossectomy defect reconstruction with its versatility in design, long pedicle with a suitable vessel diameter, and the neglectable donor site morbidity.

**Key words:**Free thin anterolateral thigh flap, free radial forearm flap, hemiglossectomy, reconstruction, morbidity.

## Introduction

Tongue cancer is the most common intraoral malignancy. Most of them occur in the anterior two-thirds of the lateral border, followed by the tongue abdomen, the base of the tongue and the tip of tongue. In most of these cases, hemiglossectomy and immediate surgical reconstruction of tongue defects are performed (1). While carrying out the repair and reconstruction, it is necessary to rebuild the function and appearance of the tongue. It is also important to reduce the damage of the donor site on both function and appearance (2,3). At present, free radial forearm (FRF) flap and free thin anterolateral thigh (ALT) flap are mainly used to reconstruct tongue defects, both of which has its own advantages and disadvantages (1,4,5).

Both of them have advantages and disadvantages. Meanwhile, with the mastering of the regional anatomy in ALT, it has been found that the ALT flap can be harvested safely and thinned to no more than 4 mm without compromising flap survival. Therefore the thin ALT flap can provide a better size match in tongue reconstruction. With a linear scar in the thigh, Moreover, this procedure guarantees a better esthetic promise with only a linear scar post operatively (6,7). In this paper, we present our preliminary experience in the reconstruction of hemiglossectomy defects with thin ALT flap in comparison with the use of FRF flap.

## Patient and Methods

The study included 46 patients with tongue carcinoma (T2-T3) in the anterior two-thirds of the lateral tongue treated from December 2009 to April 2014 in the Nangjing Stomatological Hospital, Medical School of Nanjing University. There were 30 male and 16 female patients (ratio 1.9:1), age ranging from 47 to 79 years old (mean 63.6 years). These patients underwent simultaneous hemiglossectomy and intraoral defects reconstruction with either free thin ALT flap or FRF flap. None of the patients had any resection in the mandible. Free thin ALT flap was used in 12 patients, and FRF flap was used in 34 patients. All subjects were evaluated 3 months post operatively. The vascular crisis, the speech, the swallowing function, and the donor site morbidity between these two methods were compared. The data was analyzed by Student’s t-test using SPSS V.13.0. Software (SPSS, Inc., Chicago, IL), and all *P* values that were 2-sided at a value of 0.05 were considered statistically significant. All patients gave their written informed consent. This study was approved by the Ethics Committee of the Nangjing Stomatological Hospital, Medical School of Nanjing University.

**Figure 1:**
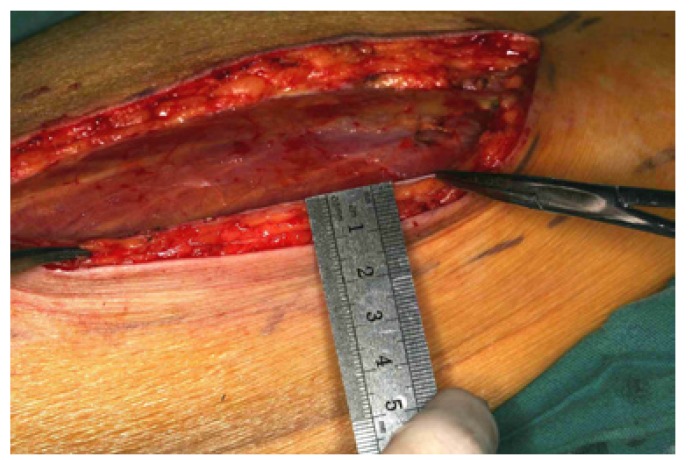
The incision was made along the medial margin of the flap down to the fascia. The ALT flap before the trimming (10 mm thick).

**Figure 2:**
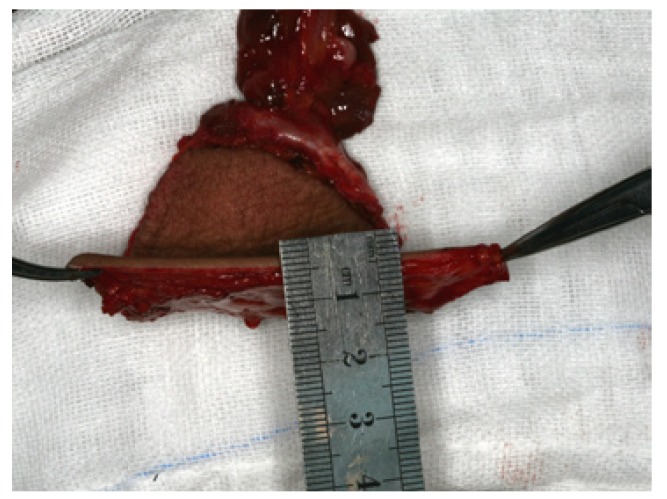
The thinning of ALT flap was completed, and it can be safely thinned to 4 mm thick.

**Figure 3:**
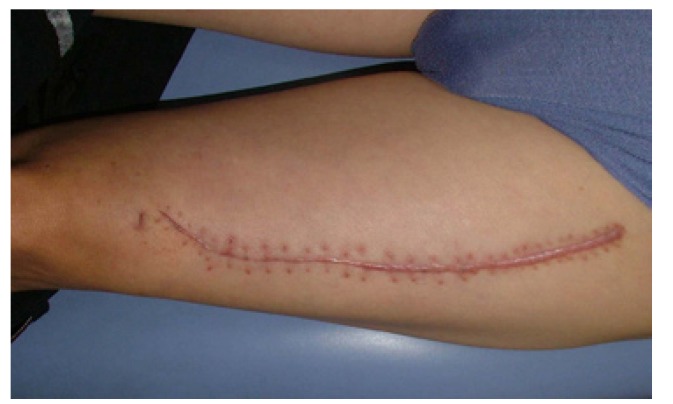
The donor-site scar of the left thigh 3 months after surgery.

- Surgical technique

1. To harvest the free thin ALT flap, firstly the descending branch of the lateral circumflex femoral artery was exposed and isolated by blunt dissection in the inter muscular septum between the rectus femoris muscle and the vastus lateralis muscle. Depending on the design of the thin ALT flap needed, the procedure required careful dissection of the septocutaneous perforator or the musculocutaneous perforator in the vastus lateralis muscle. Following the perforator via intramuscular dissection, a 0.5-cm muscle cuff around the vessel was preserved to protect the perforator. Then, the ALT flap was carefully lifted. The fascia and subdermal fat were trimmed before transplantation. Finally, the ALT flap could be safely thinned to 4 mm, and a 1.0- to 1.5-cm fascia and subdermal fat cuff around the perforator were preserved (Figs. 1,2). The donor defects were closed directly (Fig. 3).

2. To harvest the FRF flap, firstly the dissection proceeded deep the ante brachial fascia, elevating the fascia with the flap. The distal end of radial artery and venae comitans were ligated and divided. Then, the flap could be raised from the distal aspect. As the dissection proceeded proximally, the pedicle traveled under the brachioradialis tendon and muscle. Care was taken to avoid injuring a dorsal branch of the radial nerve injury. The cephalic vein was taken with the flap. Finally, the defect left by the flap was repaired with grafted skin.

3. All flaps were anastomosed to neck vessels.

- Function evaluation 

1. To evaluate swallowing function, the patient sit naturally and drank 175ml of mineral water of room temperature (25 degrees Celsius) as fast as possible. We observed the number of times the patient swallowed, and the total time taken to drink the water. So we can calculate the mean volume of each bolus, the mean duration of deglutition per bolus, and the mean volume swallowed/second (ingestion rate).

2. To evaluate speech, the patients’ voices were recorded inside a silent room using the commercially available software (Computerized Speech Laboratory [CSL] Vs99; Yang Chen Electronic Technology Company, Beijing). They were asked to sit naturally, and the microphone was positioned 5 cm from the mouth of each patient. Then, the edited material was presented to 2 speech-language pathologists and accessed by them. Finally, we performed a spectrographic assessment of the formants of the 3 vowels of Chinese (/a/, /i/ and /u/). For this assessment, the mean values of the first 3 formants (F1, F2, F3) were extracted from the most stable part of each vowel, with a duration of approximately 5 seconds, using a broad-band spectrogram generated by the CSL module with a 300 Hz filter, which is indicated to identify the sound formants (8).

## Results

**Figure 4:**
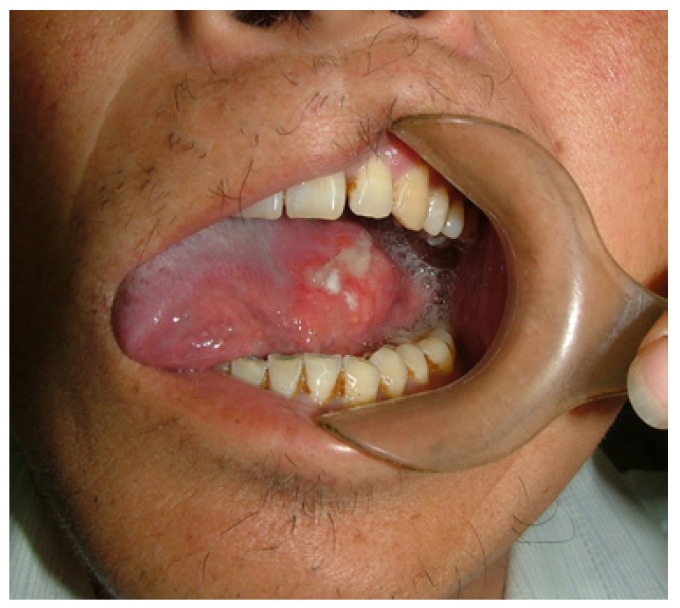
Preoperative clinical appearance of squamous cell carcinoma in the left tongue.

**Figure 5:**
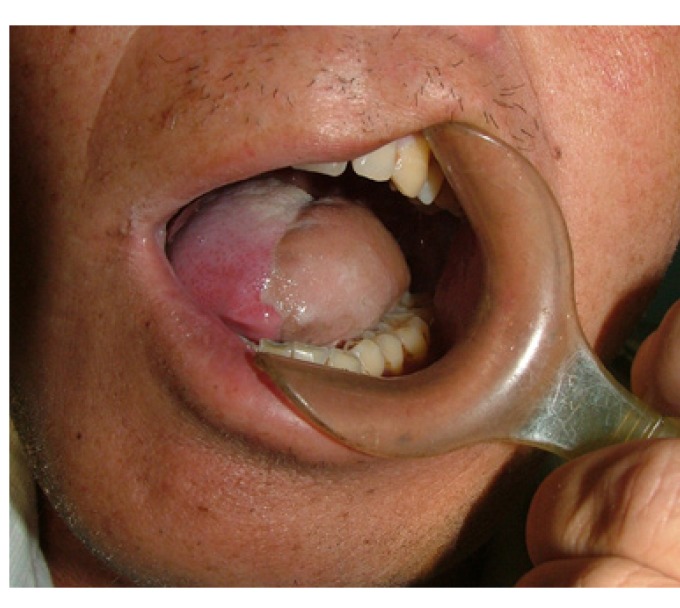
Postoperative appearance of the thin ALT flap 3 months after the tongue reconstruction.

**Figure 6:**
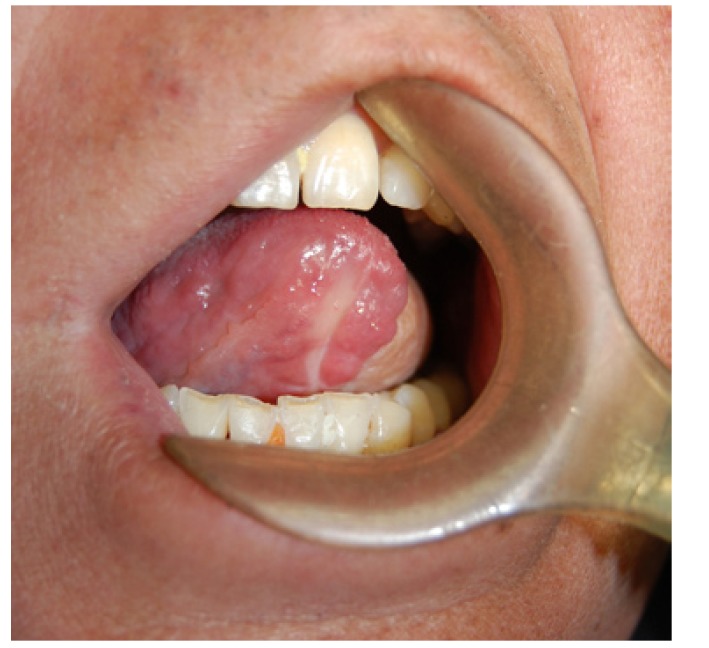
The protrusive movement of tongue after reconstruction with the thin ALT flap.

46 patients with tongue carcinoma underwent simultaneous reconstruction after hemiglossectomy. 34 patients underwent hemiglossectomy reconstruction with FRF flap; 12 patients underwent hemiglossectomy reconstruction with free thin ALT flap (Fig. 4-6). In the thin ALT flap group, all reconstruction succeeded. There was vascular crisis in 1 case, and secondary treatment was taken to rescue the flap( occurrance rate 8.3%). In the FRF flap group, 33 flaps survived, and there was necrosis in 1 flap (success rate 97.1%). There was vascular crisis in 3 cases (occurs rate 8.8%) and secondary treatment was needed. In the thin ALT flap group, the donor sites were all closed directly without complication. Meanwhile, in the FRF flap group, the donor sites were closed with grafted skin. In 4 cases, a partial grafted skin loss was observed and the donor sites healed for long time under local wound care. 9 cases suffered from fingers numbness to some degree.

Comparing the results of two methods, no statistically significant differences were found between the groups in the swallowing function (*P*>0.05) ( Table 1). Similarly, no significant differences were observed between the groups in mean speech intelligibility (reconstructed with free ALT flap: 70.62±5.18%, with FRF flap: 73.56±6.44%) (*P*>0.05). Finally, no statistically significant differences were found between the groups in F1, F2 and F3 values for the Chinese Vowels /a/, /i/ and /u/ (Student’s t-test; *P*>0.05) ( Table 2).

**Table 1:**
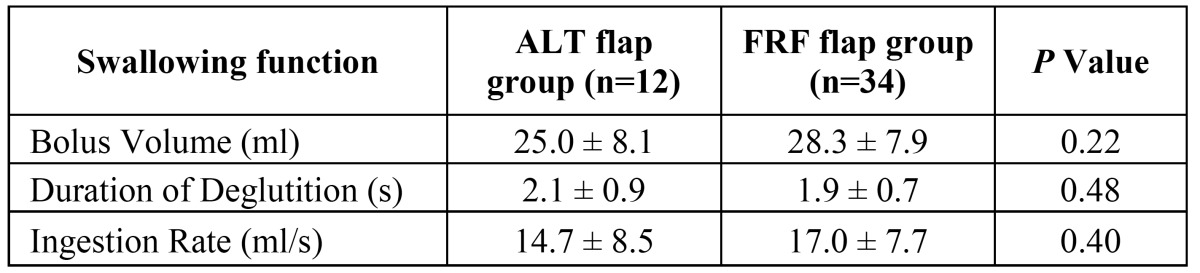
Compared swallowing function in patients after free thin ALT flap reconstruction with FRF flap.

**Table 2:**
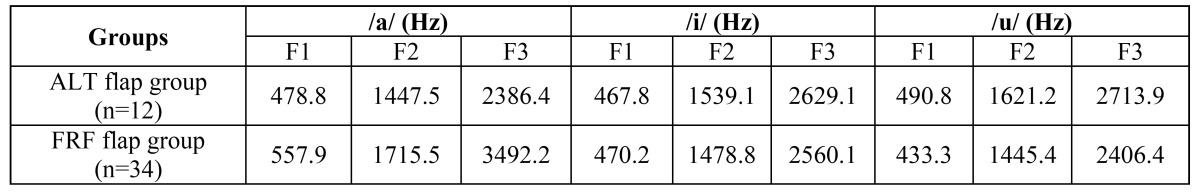
The mean values between the free thin ALT flap group and the FRF flap group (The /a/, /i/ and /u/ are Chinese Vowels.).

## Discussion

Since the first report of free ALT flap by Song *et al*. (9) in 1984, the free ALT flap has become more and more popular in body soft tissue defect reconstruction because it is reliable and versatile (10-12). However, it is usually too thick to reconstruct tongue defects. Although secondary debulking procedures can be performed (13), they impose additional burdens and stress on patients. As the reconstruction of tongue defects often requires a relatively thin skin flap, at present the most widely used flap for tongue defects is the FRF flap, which was first introduced by Yang *et al*. (14) in 1981. Among our serial patients, 34 patients underwent the reconstruction of hemiglossectomy defects with the FRF flap, and the results were acceptable. There are many advantages in reconstructing hemiglossectomy defects with the FRF flap: (1) easiness of harvest, (2) 2-team approach, (3) long pedicle, and (4) a thin and pliable skin paddle (15). However, it has some obvious disadvantages (16,17). The FRF flap requires sacrifice of the major artery of the forearm, the radial artery. The FRF flap leaves behind a conspicuous esthetic deformity in the forearm and also carries the risk of exposing important structures, such as tendons and nerves, which may result in potential dysfunction such as hand stiffness, pain, anesthesia, and poor visual appearance. In this group, partial loss of graft skin occurred in 4 cases, and 9 cases suffered from numbness of fingers to some degree. Therefore, the surgeon has always been trying to find the alternative flap to the FRF flap to reduce the surgical complications.

Since the report of thinning techniques of the ALT flap by Kimura *et al*. (6) in 1996, the thin ALT flap has become one of the preferred flaps for the reconstruction of tongue defects in some centers (18-22). For the ALT flap can be trimmed to the subdermal fat as thin as 4 mm (6,23), the thin ALT flap could provide similar soft tissue coverage as the FRF flap in the reconstruction of hemiglossectomy defects, but results in improved donor-site appearance (22-25). The donor site can be closed primarily, only leaving a linear scar, which is inconspicuous with normal clothing, and no functional deficit is left behind in the thigh. Furthermore, depending on the design of the flap needed, the thickness and the volume of the ALT flap can be adjusted for the individual extent of the defect (26). The appropriate flap bulk is also important in improving the tongue to palate contact, which is necessary for a bolus of food to descend into the hypopharynx (1). In our cases, 12 Among our patients underwent reconstruction of hemiglossectomy defects with the free thin ALT flap. The results were satisfying ( Table 1).

Tongue resections usually lead to important changes regarding swallowing function and speech. The tongue plays an essential role in speech as the main articulator of vowels. Vowels are identified by their formants, i.e., the natural resonance frequencies of the vocal tract in the articulatory position of the spoken vowel. The formant F1 values is not only related to the vertical movements of the tongue, but also influenced by closing of the mouth and narrowing of the pharynx. The formant F2 values is related to the anterior position of the tongue or to its lowering in the posterior region. The formant F3 is related to the size of the oral cavity (8,27,28).

After hemiglossectomy defect reconstruction, the changes in configuration and volume of the oral cavity generate resonant and articulatory alterations, thus intelligibility of the patient’s speech is lowered. However, very few works have been done to evaluate phonetic changes in the hemiglossectomy patients. This study compared the postoperative speech and swallowing function of 12 patients underwent free thin ALT flap reconstruction after hemiglossectomy with that of 34 patients underwent FRF flap reconstruction after hemiglossectomy, and analyzed the effects on the speech intelligibility and acoustic spectrographic characteristics of the formants of oral vowels in Chinese language, specifically the first 3 formants.

As tongue defects due to tumor radical resection vary in patients, the comparison of flaps is difficult. By restricting the patients with carcinoma of the anterior two-thirds of the lateral tongue who had as close to a hemiglossectomy as possible, Hsiao *et al*. (4,5) found no statistically significant difference in speech and swallowing function between ALT group and FRF flap group. We have the similar results. All the patients were evaluated for their functional outcome after 3-month follow-up. The evaluation included the speech, swallowing function, and donor site morbidity. Our study showed that the reconstruction with either the thin ALT flap or the FRF flap after hemiglossectomy yields quite reasonable speech and swallowing function. Although neither flap restores speech nor swallowing function to the same level as that in normal individuals, the results are still acceptable (4). Through Student’s t-test analysis, we found no statistically significant difference between two groups. The goal of hemiglossectomy defects repair is to maximize the movement of tongue and to minimize the morbidity of surgery. Therefore, we compared the incidence of vascular crisis, the speech and the swallowing function, as well as the donor site morbidity in patients underwent either ALT flap or FRF flap reconstruction after hemiglossectomy. It is reasonable to conclude that both the free thin ALT flap and the FRF flap can provide acceptable functional restoration after radical tumor resection of tongue. The ALT flap, with its versatility in design, long pedicle with a suitable vessel diameter, and low donor site morbidity, could be an ideal flap for hemiglossectomy defects reconstruction (5).
